# Supplementary figures and images for: Human cerebral spheroids undergo 4-aminopyridine-induced, activity associated changes in cellular composition and microrna expression
Source: Sci Rep. 2022 Jun 1;12:9143. doi: 10.1038/s41598-022-13071-x (PMC9160269; doi:10.1038/s41598-022-13071-x)

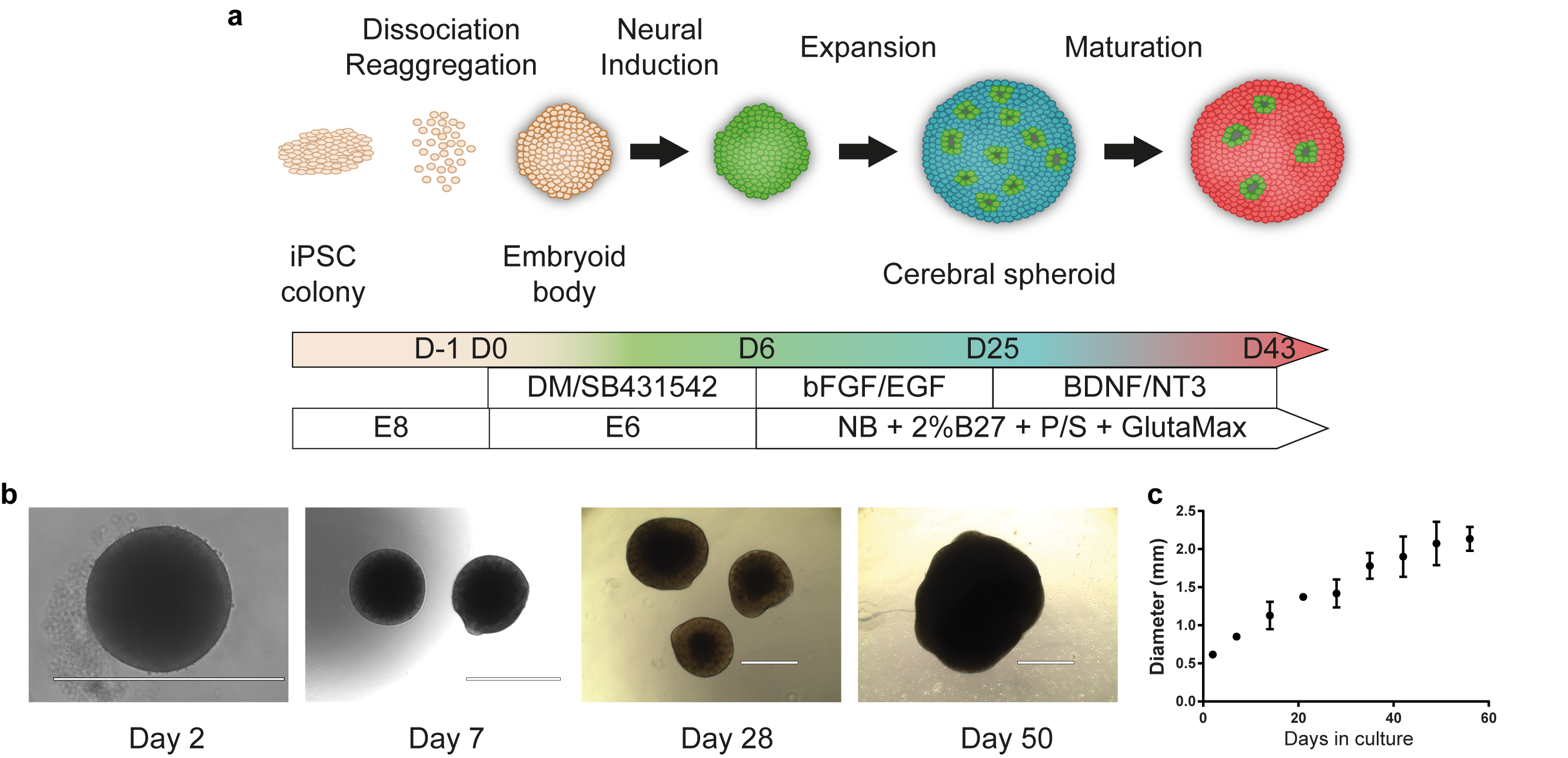

Supplement: Supplementary file 1 — Supplementary Information 1. [file 41598_2022_13071_MOESM1_ESM.tif]
