# Supplementary material for: Human cerebral spheroids undergo 4-aminopyridine-induced, activity associated changes in cellular composition and microrna expression
Source: Sci Rep. 2022 Jun 1;12:9143. doi: 10.1038/s41598-022-13071-x (PMC9160269; doi:10.1038/s41598-022-13071-x)
Supplement: Supplementary file 2 — Supplementary Information 2. [file 41598_2022_13071_MOESM2_ESM.pdf]

Supplemental information

***Supplementary Figure 1:* Overview of cerebral spheroid development**

(A) Schematic diagram of cerebral spheroid protocol. Adapted from Yoon et al., 2019.

(B) Representative bright field images over time in culture. Bar = 1 mm

BDNF: brain-derived neurotrophic factor, bFGF: basic fibroblast growth factor, DM: dorsomorphin, EGF: epidermal growth factor, iPSC: induced pluripotent stem cells, NB: Neurobasal medium, NT3: neurotrophin 3, P/S: Penicillin/Streptomycin

(C) Cerebral spheroids diameter during days in culture. Bars represent mean +/- SD. Note that for early timepoints, the error bars are smaller than the symbol. N = 4-11 spheroids/time point.

<u>Supplementary Table 1: Primary and secondary antibodies used</u>

**Antigen**	**Host species**	**Working dilution**	**Manufacturer (catalog number)**
Sox2	Rabbit	1:500	Abcam (ab97959
Nestin	Rabbit	1:500	Abcam (ab92391)
DCX	Rabbit	1:500	Abcam (ab18723)
Map2	Mouse	1:200	Millipore (MAB3418)
NeuN	Mouse	1:500	Millipore (MAB377)
GFAP	Rabbit	1:1000	Millipore (AB5804)
Ki67	Rabbit	1:500	ThermoFisher (PA5-19462
Cleaved Caspase 3	Rabbit	1:500	Cell signaling technologies (9661T)
cFOS	Rabbit	1:500	Cell signaling technologies (4384)
Anti-Rabbit IgG Alexa Fluor 488	Donkey	1:500	Invitrogen (A-21026)
Anti-Mouse IgG Alexa Fluor 594	Goat	1:500	Invitrogen (A-11032)

Supplementary Table 2: PCR primers used:

**Target**	**Sequence**	**Efficiency**	**Reference**
Human GAPDH	Fwd: TGCACCACCAACTGCTTAGC<br>Rvse: GGCATGGACTGTGGTCATGAG	110%	Primer BLAST
Human HPRT	Fwd: AGCTTGCTGGTGAAAAGGAC<br>Rvse: TTATAGTCAAGGGCATATCC	105.8%	Primer BLAST
Human RMST	Fwd: GCAGTGGGTGACTGATCGTA<br>Rvse: AGTCAACTCCGTGTCCCTTG	97.7%	Primer BLAST
miR-125b	CTCCCTGAGACCCTAACTTGTG	94.6%	IDTQuanta
miR-132	CAGTCTACAGCCATGGTCGAAA	109.3%	IDTQuanta
miR-135a	GCTATGGCTTTTTATTCCTATGTGA	106.9%	IDTQuanta
miR-139	CAGTGCACGTGTCTCCAGTAAAA	108.5%	IDTQuanta
miR-146a	TGAGAACTGAATTCCATGGGTTA	130.9%	IDTQuanta
miR-17	CCACAAAGTGCTTACAGTGCAG	108.7%	IDTQuanta
miR-181c	AACATTCAACCTGTCGGTGAGT	107.4%	IDTQuanta
miR-19a	TGTGCAAATCTATGCAAAACTGA	103.6%	IDTQuanta
miR-21	GCTAGCTTATCAGACTGATGTTGAAA	106.1%	IDTQuanta
miR-30a	CGATGTAAACATCCTCGACTGG	104.6%	IDTQuanta
miR-9	CGCTCTTTGGTTATCTAGCTGTATG	97.2%	IDTQuanta
Pri-miR-135a1	Fwd: CCTCGCTGTTCTCTATGGCTTT<br>Rvse: ACGGCTCCAATCCCTATATGA	102.3%	Primer BLAST
Pri-miR-135a2	Fwd: TCACTCTAGTGCTTTATGGCTT<br>Rvse: TGGCTTCCATCCCTACATGA	101.4%	Primer BLAST
Universal primer	GCATAGACCTGAATGGCGGTA		IDTQuanta
